# Poor quality of life as a predictor of survival among thalassemia patients in Iran

**DOI:** 10.4178/epih.e2017013

**Published:** 2017-03-28

**Authors:** Shahab Rezaeian

**Affiliations:** Research Center for Environmental Determinants of Health (RCEDH), Kermanshah University of Medical Sciences, Kermanshah, Iran

**Dear Editor,**

An Iranian study was recently published in your prestigious journal, in which the aim was to determine the association between health-promoting lifestyles and quality of life (QoL) among a sample of 389 adult beta-thalassemia major (TM) patients [1]. The findings of that study revealed an unacceptable level of QoL among the thalassemia patients, in accordance with another finding from an Iranian study [2,3]. So far, none of the studies conducted on the survival rate of patients with thalassemia [4,5] have addressed the effect of QoL on survival in the discussion section.

We conducted a retrospective cohort study enrolling 704 TM patients in 2016 in Shiraz, southern Iran. Our study aimed to identify changes in the survival rate of TM patients over the past two decades. Two different approaches, including a cohort analysis (Figure 1) and a period analysis (Table 1), were used to evaluate 20-year and 30-year survival rates and to compare changes in the survival trends in various time intervals between 1995 and 2016. The Cochran-Armitage test was used for trend analysis.

A downward trend was found in both the 20-year (p< 0.001), and 30-year (p< 0.001) survival rates of TM patients from 1995 to 2007, but an upward trend was found from 2007 through 2016 (Figure 1). The period analysis also indicated a downward trend in 20-year (p= 0.002), and 30-year (p= 0.005) survival rates from 1995 to 2016 (Table 1).

With these results in mind, the poor QoL reported among Iranian TM patients [1-3] could explain the downward trend in the survival rate of TM patients found in this study. The results of a study conducted to characterize health-related QoL (HRQoL) and its related factors found that chronic diseases, such as thalassemia, were a significant determinant of HRQoL [6]. Another important factor related to QoL and life satisfaction is self-rated health status, which has been studied in the general population [7] and among subjects with a chronic illness [8].

In conclusion, it is suggested that factors affecting QoL should be addressed in future studies to obtain important information for policymakers to improve the QoL in this population, thereby improving survival.

## Figures and Tables

**Figure 1.**
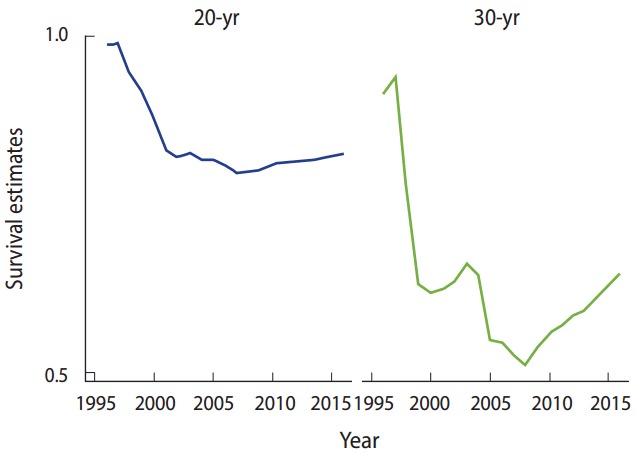
The 20-, and 30-year survival rates of thalassemia major patients obtained at various time intervals, Iran, 1995-2016.

**Table 1.** Trends in the 20- and 30-year survival rates of thalassemia major patients by period analysis, Iran, 2016

	Period				p-value
	1995-2000	2001-2005	2006-2010	2011-2016	
No. of subjects	196	378	519	597	-
Deaths	12	39	60	66	-
Mean probability of 20-year survival	0.94	0.82	0.80	0.82	0.002
Mean probability of 30-year survival	0.77	0.62	0.53	0.61	0.005
